# Molybdenum Sulfide (MoS_2_)/Ordered Mesoporous Carbon (OMC) Tubular Mesochannel Photocatalyst for Enhanced Photocatalytic Oxidation for Removal of Volatile Organic Compounds (VOCs)

**DOI:** 10.3389/fchem.2021.748622

**Published:** 2022-01-28

**Authors:** Li He, Wei Guan, Yao Zeng, De Zhao, Xuemin Qiu, Guo Jia

**Affiliations:** ^1^ Resource and Environment College, Zunyi Normal University, Zunyi, China; ^2^ Chongqing Key Laboratory of Environmental Materials and Remediation Technologies, Chongqing University of Arts and Sciences, Chongqing, China; ^3^ Ecological Environment Monitoring Station of Dadukou District, Chongqing, China; ^4^ College of Mathematics and Statistics, Chongqing University, Chongqing, China; ^5^ Chongqing Jingchuang United Environmental Protection Engineering Co. Ltd, Chongqing, China

**Keywords:** volatile organic compounds, formaldehyde, molybdenum sulfide, ordered mesoporous carbon, composite photocatalyst

## Abstract

Air pollutants cause severe problems in urban areas, specifically in terms of toxicological impacts on human health. Therefore, it is urgent to develop an effective, safe, and inexpensive technique to remove these air pollutants. In this study, the MoS_2_/OMC heterojunction photocatalyst was successfully synthesized by a hydrothermal process and used for improving photocatalytic oxidation performance for removing VOCs. Formaldehyde was used as a model VOC in the gas phase and was removed at room temperature under visible light irradiation. For my research, the microstructure and morphology of the photocatalyst were deeply characterized, and the results indicated that MoS_2_ were successfully coupled into OMC materials to prepare MoS_2_/OMC heterojunction photocatalysts. The trend of photocatalytic efficiency for formaldehyde decomposition was MoS_2_/OMC > MoS_2_ > OMC. Besides, the MoS_2_/OMC heterojunction photocatalyst showed an excellent regeneration performance after several recycles, indicating the potential of MoS_2_/OMC composite as a promising photocatalyst for VOC removal. These results indicated that the photocatalytic reactor containing MoS_2_/OMC photocatalysts was highly active and stable.

## Introduction

Recently, indoor air quality has a very important impact on human health and comfortable life, and the bad indoor air quality can lead to physical discomfort, poor health, and a variety of diseases (Lim et al., 2021). Volatile organic compounds (VOCs) among these indoor air pollutants are difficult to control and harmful to human body (Bunch et al., 2014; Chen et al., 2021). Besides, VOCs have been proven to be related to a variety of human diseases, and some of them even have genotoxicity and carcinogenicity, which critically harm human health (Shayegan et al., 2019). VOCs include formaldehyde, benzene, toluene, low alcohol, ketone, and chlorine-containing organics, mainly resulting from paints, coatings, solvents, preservatives, air fresheners, and disinfectants (Huang et al., 2021a). Formaldehyde is one of the main indoor organic pollutants affecting human health; therefore, efficient removal technology for detection and control of VOCs in the indoor environment has attracted much more attention (Zeng et al., 2020; Huang et al., 2021b).

It is imperative to control the emission of VOCs from the source, and more efforts have been made to develop efficient technologies to resolve the problem of VOC elimination, such as biodegradation (Wantz et al., 2021), adsorption (Li et al., 2021b), and catalytic oxidation (Guo et al., 2021). The toxicity of VOCs limits the degradation effect of VOCs using biodegradation technology (Zou et al., 2019). Adsorption technology is considered to be the most economical and promising control strategy, especially for low concentration of VOCs, which is a simple operation at relatively low cost (Zhu et al., 2020). Catalytic oxidation has limited its application because of its high operating temperature and cost (Liu et al., 2021). In recent years, photocatalytic oxidation technology mainly produces reactive oxygen species on the surface of the catalyst by light and achieves the degradation of contaminant and hydrogen evolution (Guan et al., 2018; Zada et al., 2020; Zhang et al., 2021d).

Recently, TiO_2_ is the most widely studied and applied photocatalytic material, but the key problems limiting its large-scale application are low efficiency of photocatalytic degradation of VOCs and large bandgap width (Suárez et al., 2019; Tobaldi et al., 2021). To make most use of solar energy, researchers have developed a series of new and highly efficient photocatalytic materials that respond to visible light (Shi et al., 2019; Wang et al., 2020). Liao et al. reported that the remodeling of the g-C_3_N_4_ p-n homojunction with nitrogen vacancies is a feasible way as highly efficient photocatalysts (Liao et al., 2021). Polymetallic sulfides have attracted much attention because of their narrow bandgap and suitable valence band conduction potential, such as ZnIn_2_S_4_ (Zhang et al., 2021b), CdIn_2_S_4_ (Liu et al., 2019a), and CaIn_2_S_4_ (Xia et al., 2017). For single semiconductor photocatalysts, the lower photocatalytic efficiency is usually due to its high recombination probability of photogenerated carriers (Xia et al., 2016; Mei et al., 2021), while composite photocatalysts can solve this problem by promoting the effective separation of photogenerated carriers through the interface effect (Wang et al., 2019; Qi et al., 2020). Molybdenum disulfide (MoS_2_), a kind of metal sulfides and good photocatalytic material, is similar to graphene with a two-dimensional layer structure. The relatively weak van der Waals force between the layers shows many excellent properties, such as high electron mobility, specific surface area, and unsaturated surface structure (Tian et al., 2020b; Huang et al., 2020). Besides, two-dimensional optoelectronic functional materials as novel semiconductor photocatalysts have attracted extensive attention from researchers. MoS_2_ have been applied broadly in photocatalysis such as water splitting (Wang et al., 2009; Dong et al., 2020) and degradation of organic pollutant (Monga et al., 2020; Zhang et al., 2021a). The preparation of MoS_2_ with other semiconductor materials was used to improve the photocatalytic activity by forming heterostructures.

The photocatalytic reaction rate mainly depends on the reaction rate constant and adsorption coefficient (Hasija et al., 2020). When the adsorption process is the rate-limiting step of the photocatalytic reaction, improving the adsorption performance of the photocatalyst can improve the photocatalytic reaction efficiency (Li et al., 2021a). Combining the photocatalytic activity of MoS_2_ with the adsorption performance of the adsorbent, the organic matter adsorbed on the adsorbent can be degraded through photocatalytic action, so as to enhance the purification ability of the adsorbent and prolong the service life of the adsorbent (Zhou et al., 2021). The adsorption capacity of the adsorbent carrier provides a high concentration environment for photocatalytic reaction, which can improve the efficiency of photocatalytic reaction. Therefore, it can further make the photocatalytic technology to improve its superior performance of removing harmful gases of VOCs. Liu et al. reported the 3D Pd@MoS_2_-conjugated polypyrrole frameworks, which demonstrated that the Tsuji–Trost reaction using Pd@MoS_2_CPFs photocatalysts could be realized under visible light and with improved photocatalytic performance (Liu et al., 2019b). Zhang et al. reported that P-doped MoS_2_/g-C_3_N_4_ not only generated a dual IEF to drive charge migration but also facilitated spatially separated redox sites to further promote the separation of photo-induced carrier (Zhang et al., 2022). Ordered mesoporous carbon (OMC) materials have the characteristics of high specific surface area, large pore volume and uniform pore size distribution, good thermal stability, good electrical conductivity, high mechanical strength, and good chemical inertia, which acts as a good adsorbent for contaminant removal (Tian et al., 2020a; Tian et al., 2021).

Herein, the MoS_2_/OMC heterojunction photocatalyst was successfully synthesized by a hydrothermal process and used for improving photocatalytic oxidation performance for VOC decomposition. Formaldehyde was used as model VOCs in the gas phase and was removed at room temperature under visible light irradiation. The microstructure and morphology of MoS_2_/OMC photocatalyst were deeply characterized, and the optimal conditions for formaldehyde removal were analyzed. The separation mechanism of photogenerated electrons and holes of MoS_2_/OMC photocatalysts were investigated. Moreover, the stability performance of the MoS_2_/OMC heterojunction photocatalyst was deeply analyzed after several recycles. Last, the possible photocatalytic mechanism of formaldehyde removal using heterojunction photocatalyst was proposed.

## Materials and Methods

### Chemicals

Formaldehyde (HCHO), sodium molybdate dihydrate (Na_2_MoO_4_ 2H_2_O), thiourea (NH_2_CSNH_2_), N,N-dimethylformamide (C_3_H_7_NO), ethanol absolute (C_2_H_6_O), and isopropanol (C_3_H_8_O) were bought from Sinopharm Chemical Reagent Co., Ltd (Beijing, China), and 5, 5-dimethyl-1-pyrroline-N-oxide (DMPO) used for radical analysis was provided by Sigma Chemical Co. Ltd. Ordered mesoporous carbon (OMC) was provided by Low-Dimension Materials. The type of OMC was CMK-3 and provided by the Low-Dimension Materials. The specific surface area was 1,000 m^2^/g, total pore volume was 1.35 cm^3^/g, and the micropore volume was 0.01 cm^3^/g. All chemicals used in the experiments were analytically pure and provided by the company without further purification.

### Preparation of MoS_2_/OMC Photocatalysts

The MoS_2_/OMC heterojunction photocatalyst was successfully synthesized by a hydrothermal process (Bian et al., 2012). Sodium molybdate dihydrate and thiourea were used as precursors to prepare the MoS_2_/OMC photocatalyst. A total of 100 mg OMC was added to C_3_H_7_NO solution and then mixed the solution with Na_2_MoO_4_ 2H_2_O (0.15 g) and NH_2_CSNH_2_ (0.24 g) by ultrasonication; the mixed solution was then transferred to a stainless steel high-pressure reactor, followed by the hydrothermal reaction at 200°C for 24 h. Finally, the black precipitate was collected by centrifugation after naturally cooling to room temperature and washed with deionized water and ethanol. Subsequently, the precipitate was dried at 80°C for 24 h under vacuum and calcined for 2 h, noting as MoS_2_/OMC photocatalyst.

The pure MoS_2_ materials were prepared as follows: Na_2_MoO_4_ 2H_2_O (0.3 g) and NH_2_CSNH_2_ (0.8 g) were mixed well into distilled water (65 ml) by ultrasonication and then the mixed solution was transferred to a stainless steel high-pressure reactor, followed by the hydrothermal reaction at 200°C for 24 h. The black precipitate was collected by centrifugation after naturally cooling to room temperature and washed with deionized water and ethanol. The precipitate was dried at 80°C for 24 h under the vacuum and calcined for 2 h, and after that, the prepared powders were further grinded into smaller powders with a mortar.

### Characterization

The crystal phase structure and mesoporous order degree of photocatalysts were analyzed by using an X-ray diffraction (XRD) instrument (Bruker, D8 ADVANCE). The scanning speed was 0.05°/s, and the scanning range of 2θ was 10°–90°. The microscopic morphology of the material was characterized by scanning electron microscopy (SEM) (S-4800). The microstructure of the photocatalysts was further analyzed with a transmission electron microscope (TEM, Phillips model CM200). The physical adsorption properties of the materials were measured by a Micromeritics ASAP 2010 adsorption apparatus. Besides, photocurrent analysis was performed with an electrochemical workstation (CHI 660D).

### Analysis of Photocatalytic Performance

The top of double-layer glass reactor (300 ml) was sealed by a quartz sheet, and the outer interlayer was refluxing water in the photocatalytic reaction process to ensure the photocatalytic reaction was carried out at room temperature. The excitation light source used in the reaction was a 300 W xenon lamp, supported with a UV420 filter (PLS-SXE300D, Beijing), ensuring the excitation light source in the process of the photocatalytic reaction was visible light (420 nm≤*λ* ≤ 780 nm). The photocatalyst powder was uniformly dispersed in a petri dish (diameter 6 cm) containing 1 g of ethanol absolute under ultrasonication and then dried at 40°C.

The petri dishes loaded with photocatalysts were placed at the inner bottom of the double-layer glass reactor, and the visible light source excited by the xenon lamp was illuminated to the surface of the petri dishes through the quartz window at the top. The distance between the petri dishes and the UV420 filter was 10 cm. The reactor was purged with 60 ml/min of high-purity nitrogen for 45 min to eliminate CO_2_, eliminating VOCs and other gases in the reactor and gas pipeline before the reaction. During the photocatalytic reaction, a gas-tight syringe (SGE, 500 μL) was used to collect reaction gas at intervals through the acquisition window of the double-layer glass reactor, and the reaction gas was detected by using a gas chromatograph. The removal efficiency (η) of formaldehyde under visible light irradiation was calculated by the following equation:

$$\text{η}=\frac{{\text{C}}_{0}-{\text{C}}_{\text{t}}}{{\text{C}}_{0}}\times 100\text{\%,}$$ (1)

where C_t_ and C_0_ are the concentrations of formaldehyde in the reactor at each given time and initial time, respectively.

## Results and Discussion

### Material Characterization

**FIGURE 1:**
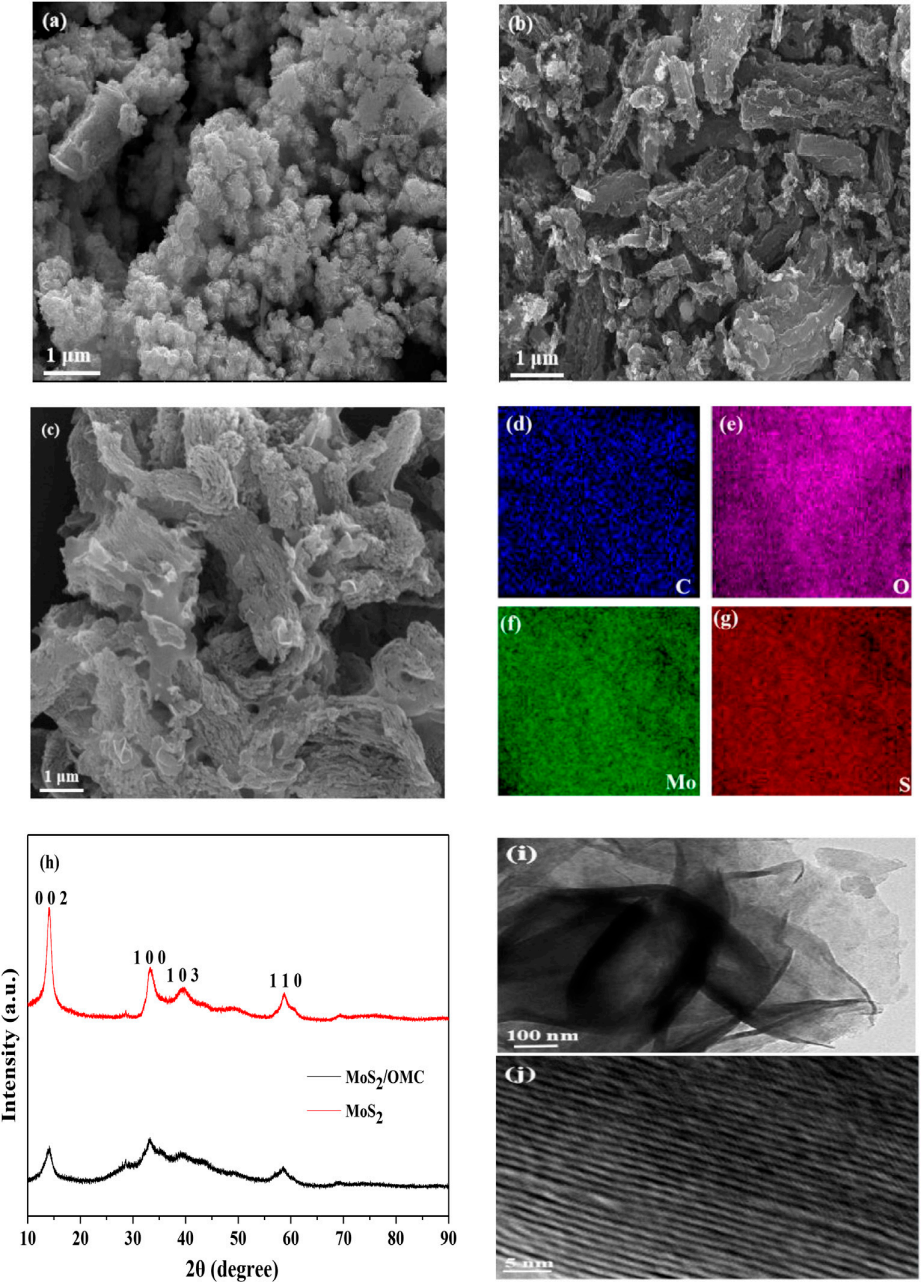
SEM analysis of materials: **(A)** MoS_2_, **(B)** OMC, and **(C)** MoS_2_/OMC photocatalysts; elemental maps of MoS_2_/OMC composite: **(D)** carbon, **(E)** oxygen, **(F)** molybdenum, **(G)** sulfur, **(H)** XRD analysis of MoS_2_ and MoS_2_/OMC, **(I)** TEM image of MoS_2_/OMC photocatalysts, and **(J)** HRTEM image of MoS_2_/OMC photocatalysts.

The morphology of photocatalysts (MoS_2_, OMC, and MoS_2_/OMC) was analyzed by SEM and XRD. As shown in Figure 1A, many hollow flowers structure could be observed for the pure MoS_2_ photocatalysts. As shown in Figures 1A,B, the micron-scale three-dimensional structure of OMC materials contained many pores. According to the definition of the International Union of Pure and Applied Chemistry based on pore width (usually pore diameter or layer distance of slit shape pores) (Han et al., 2019), pores in the materials can be classified into macropores (pore size >50 nm), mesopores (2 nm < pore size <50 nm), and micropores (pore size <2 nm). The pore size of OMC was 5.57 nm, and lots of mesopores were present in the OMC materials. Therefore, the pore structure of OMC had a larger specific surface area and more edge active sites (Zhang et al., 2021c). As shown in Figure 1C, the flower structure of MoS_2_ was successfully assembled into the surface of OMC. The particular structure contributed to the full contact between the degradable compounds and the catalytic active sites, which not only maintained the high activity of photocatalysts but also avoided agglomeration (Ismael, 2021). Corresponding elemental mapping analysis is shown in Figures 1D–G; the homogeneous distribution of C, O, Mo, and S elements can be observed for MoS_2_/OMC. Besides, the XRD spectra of MoS_2_ and MoS_2_/OMC are shown in Figure 1H. Comparing with the standard card, the corresponding crystal plane of the diffraction peak was (002), (100), (103), and (110), in accordance with the hexagonal structure of MoS_2_ (Wei et al., 2022). The peak of MoS_2_/OMC was observed at 2θ = 24.8° on the (002) crystal surface of OMC (JCPDS75-1621), indicating that the mesoporous carbon in the complex was amorphous carbon material after hydrothermal treatment. In order to better demonstrate the heterojunction microstructure, the TEM technique was used to analyze the surface morphology of MoS_2_/OMC photocatalysts. As shown in Figures 1I,J, MoS_2_/OMC showed an ordered nanoflower structure composed of ultrathin nanosheets with an expanded interlayer spacing, with an interplanar spacing of 0.82 nm indexed as (002) plane of MoS_2_.

**FIGURE 2:**
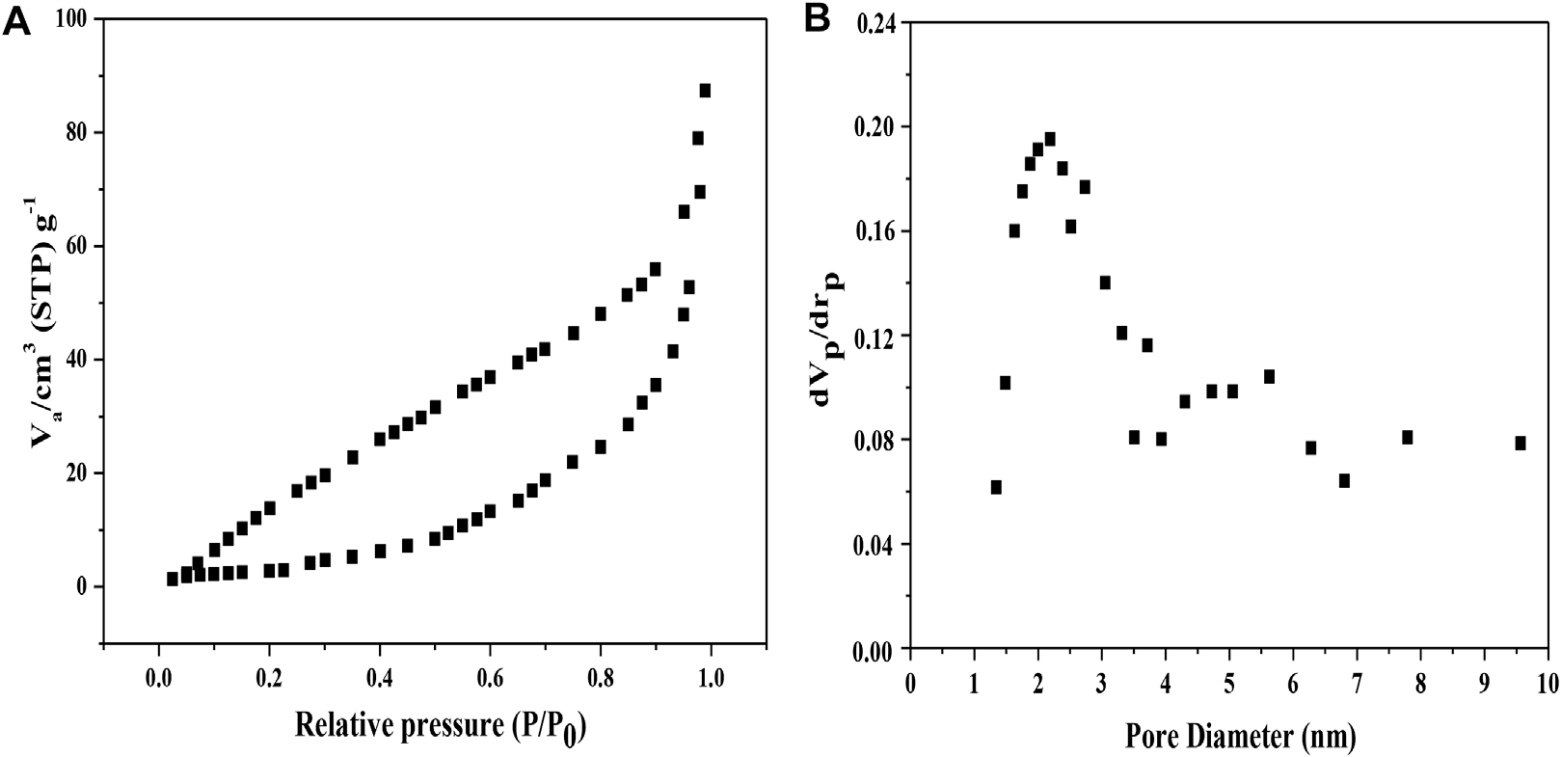
**(A)** Isotherms of nitrogen adsorption and desorption and **(B)** BJH adsorption pore size distribution of MoS_2_/OMC.

BET analysis and the hole diameter distribution of MoS_2_/OMC photocatalyst were evaluated. The N_2_ adsorption isotherms of the MoS_2_/OMC photocatalyst was type IV based on IUPAC classification, showing the existence of both micro- and mesopores (Figure 2A) in accordance with the result of pore size distributions (Figure 2B). The introduction of MoS_2_ would affect the microstructures of OMC and change the pore textures of OMC. Before modification, the specific surface area for OMC was 1,000 m^2^/g and the total pore volume was 1.35 cm^3^/g, while the calculated specific surface area of the MoS_2_/OMC photocatalyst was 613 m^2^/g after MoS_2_ decoration. As a result, MoS_2_ decoration for preparing the MoS_2_/OMC photocatalyst might block the mesopores of OMC to some extent. MoS_2_ attached on the surface of OMC could hinder the agglomeration of MoS_2_/OMC with a structure with thinner nanosheets and smaller size. Mesoporous carbon materials had a good surface chemical inert stability and mechanical strength, and their high specific surface area and uniform pores could also provide abundant reaction sites for improving the photocatalytic performance (Qiu et al., 2021).

### Optical Property Analysis

**FIGURE 3:**
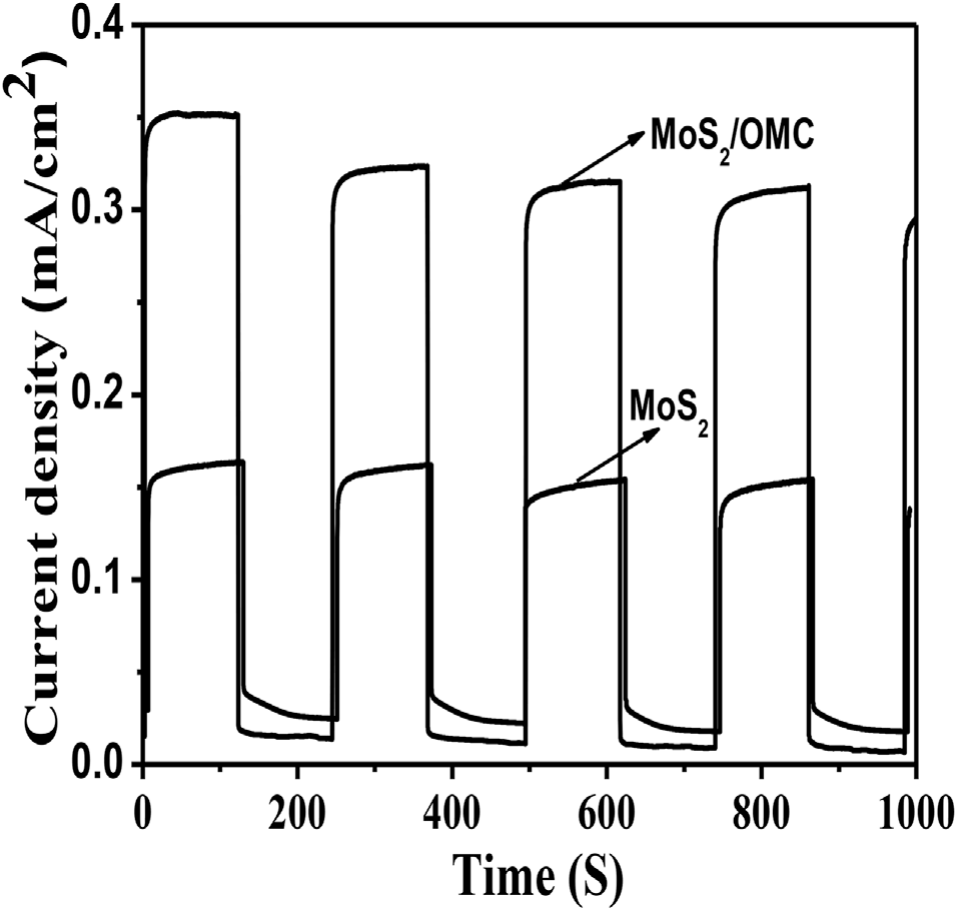
Photocurrent spectroscopy of three photocatalysts materials (MoS_2_, OMC, and MoS_2_/OMC).

For the pure MoS_2_ sample, the onset edge of the absorption peak is about at 760 nm, corresponding to a bandgap of 1.63 eV (Zhou et al., 2013). Therefore, it was urgent to prepare the micro-heterojunction structure to improve the photocatalytic activity. Photocurrent spectroscopy was used to detect the generation and separation of photogenerated electrons. As shown in Figure 3, compared with pure MoS_2_, the photocurrent density of the MoS_2_/OMC was increased, showing that the introduction of OMC increased the separation rate of photogenerated electron–hole. The photocurrent result confirmed that the heterostructure promoted the separation of photo-induced charges efficiently and improved the photocatalytic performance.

### Photocatalytic Performance Analysis

**FIGURE 4:**
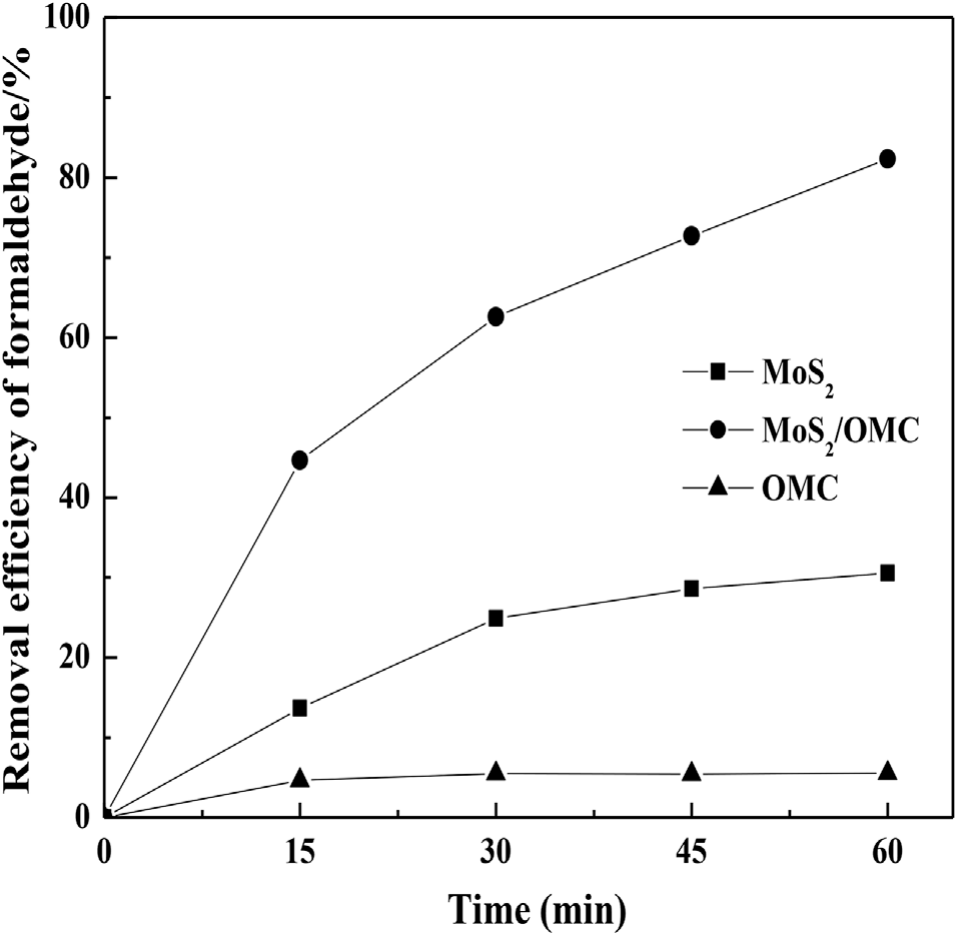
Comparison of three photocatalysts materials (MoS_2_, OMC, and MoS_2_/OMC) for formaldehyde removal.

The photocatalytic performances of different photocatalysts (MoS_2_, OMC, and MoS_2_/OMC) were analyzed. As shown in Figure 4, MoS_2_/OMC showed the obviously increased photocatalytic performances under visible light irradiation (*λ* > 420 nm) in comparison to the pure MoS_2_ and OMC (the removal efficiency of formaldehyde for MoS_2_, OMC, and MoS_2_/OMC were 30.59, 82.34, and 5.54%). The modified MoS_2_/OMC photocatalysts effectively inhibited the recombination of photogenerated electrons and photogenerated holes and prolonged the life of photogenerated carriers. Therefore, the heterojunction microstructure of MoS_2_/OMC photocatalysts was a benefit for increasing the removal efficiency of formaldehyde.

**FIGURE 5:**
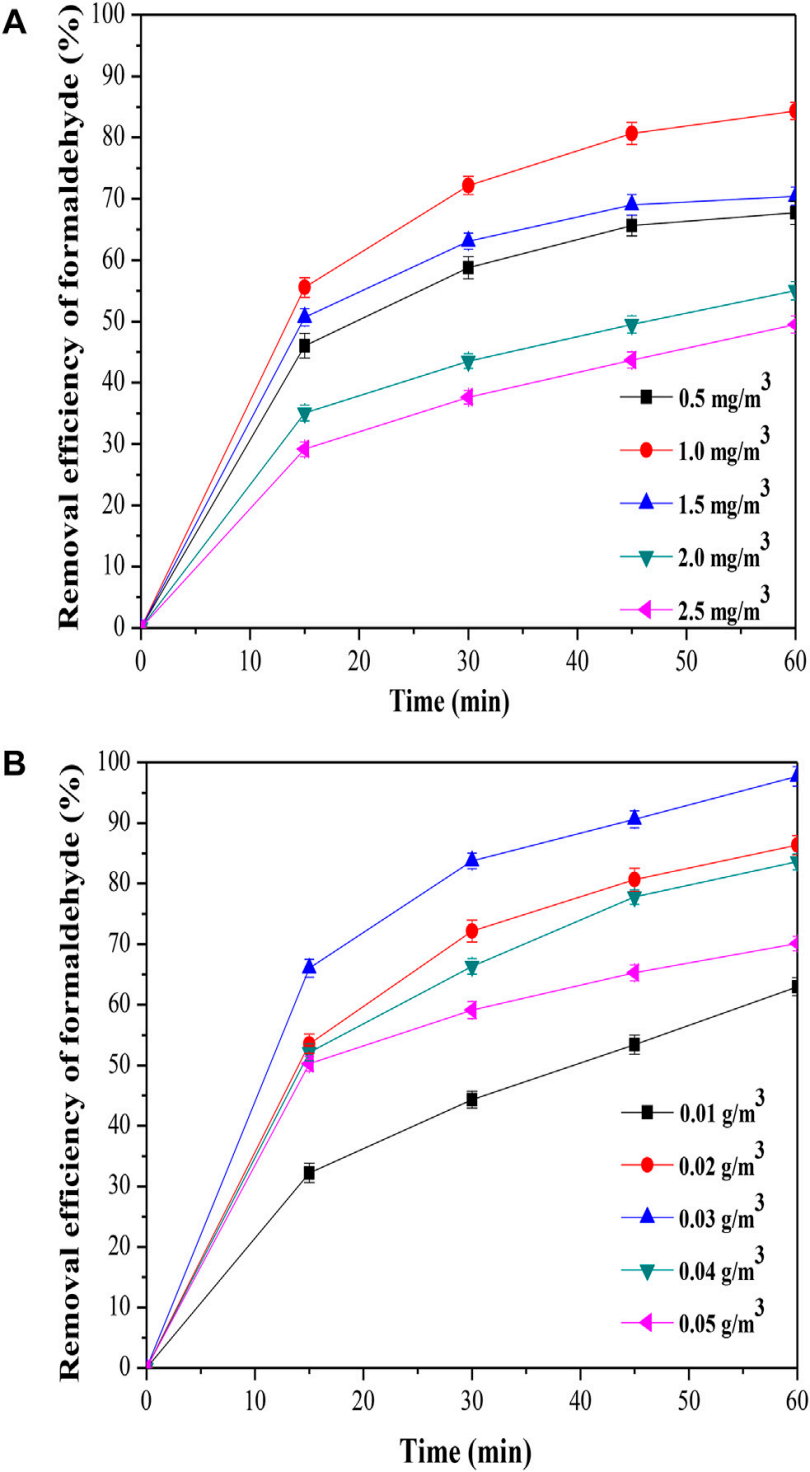
Parameter optimization analysis: **(A)** Effect of initial formaldehyde concentration on formaldehyde removal; and **(B)** effect of photocatalyst amount on formaldehyde removal.

The effect of initial concentration on formaldehyde removal was shown in Figure 5A, the removal efficiency of formaldehyde was improved from 67.72 to 84.34% when the initial concentration on formaldehyde was increased from 0.5 to 1.0 mg/m^3^. The result indicated that the active sites of the MoS_2_/OMC surface were not fully utilized with the lower concentration of formaldehyde, and the higher concentration of formaldehyde was benefited for increasing the utilization rate of MoS_2_/OMC. When the initial concentration of formaldehyde was low, the catalytic behavior of photocatalysts was mainly affected by the mass transfer and diffusion from the main gas phase to the surface of photocatalysts. The increasing initial concentration of formaldehyde increased the partial pressure and thus accelerated the reaction rate. But, the removal efficiency of formaldehyde was reduced when the formaldehyde concentration increased from 1.0 to 2.5 mg/m^3^; therefore, the optimal formaldehyde concentration for formaldehyde removal using MoS_2_/OMC as photocatalysts was 1.0 mg/m^3^. The mass transfer diffusion from the main body of the gas phase to the surface of the catalyst accelerated the formaldehyde adsorbed by the active site on the MoS_2_/OMC photocatalyst surface when the formaldehyde concentration was higher. Meanwhile, the effects of mass transfer diffusion and adsorption of formaldehyde were obviously weakened, and the catalytic reaction of formaldehyde on the surface of MoS_2_/OMC photocatalysts had become the main factor affecting the reaction.

The amount of photocatalysts was an important factor to determine the formaldehyde removal. In this experiment, the effect of photocatalysts amount on formaldehyde removal is shown in Figure 5B. The removal efficiency of formaldehyde improved gradually when the photocatalyst amount was increased from 0.01 to 0.03 g/m^3^. So the increasing amount of photocatalysts could increase the active site of the reaction, which could promote the catalytic reaction. However, the removal efficiency of formaldehyde reduced when the photocatalyst amount was increased from 0.03 to 0.05 g/m^3^. The further increase in the photocatalyst amount inhibited the removal efficiency of formaldehyde improving due to the agglomeration phenomenon and low mass transfer efficiency.

### Proposed Mechanism

**FIGURE 6:**
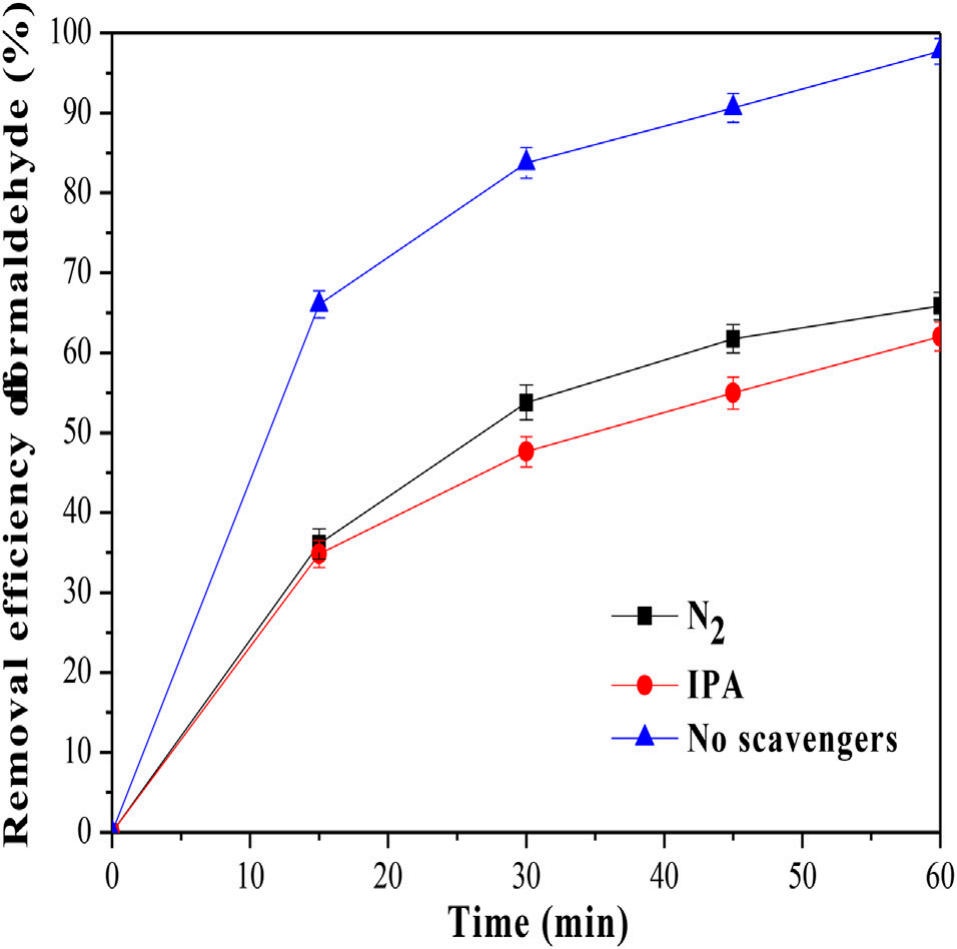
Effect of scavengers on the formaldehyde removal in the photocatalytic system.

Radical quenching experiments were further conducted to confirm the generated radicals responsible for formaldehyde removal. Isopropanol (IPA) worked as the OH radical quencher, and N_2_ gas was used to reduce the O_2_ **·** ^**-**^ radicals (Zhang et al., 2017). As shown in Figure 6, the removal efficiency of formaldehyde was decreased to 62.04% when adding IPA to the reaction solution. Furthermore, when N_2_ was bubbled into the reaction solution, the removal efficiency of formaldehyde was even decreased to 65.84%. The results indicated that both OH and O_2_^**-**^ radicals were the major active radical species for formaldehyde removal in the photocatalytic process. The photocatalytic mechanism for formaldehyde removal using MoS_2_/OMC photocatalysts was explained. During the photocatalytic process, the photogenerated electrons would react with the adsorbed O_2_ molecules on the surface of MoS_2_/OMC photocatalysts to produce O_2_ **·** ^**-**^, and the photogenerated holes would react with the adsorbed H_2_O molecules on the surface to generate OH (Lan et al., 2018). These reactive oxygen species would further react and oxidize the adsorbed HCHO molecules on the surface into CO_2_ and H_2_O.

Mesoporous OMC materials had a high specific surface area, and uniform pores could also provide abundant reaction sites for improving the photocatalytic permanence. Besides, the MoS_2_ hollow flowers uniformly grew on the surface of OMC through the hydrothermal process, reducing the agglomeration for MoS_2_/OMC photocatalysts and enhancing the photocatalytic permanence.

### Stability Performance of MoS_2_/OMC

**FIGURE 7:**
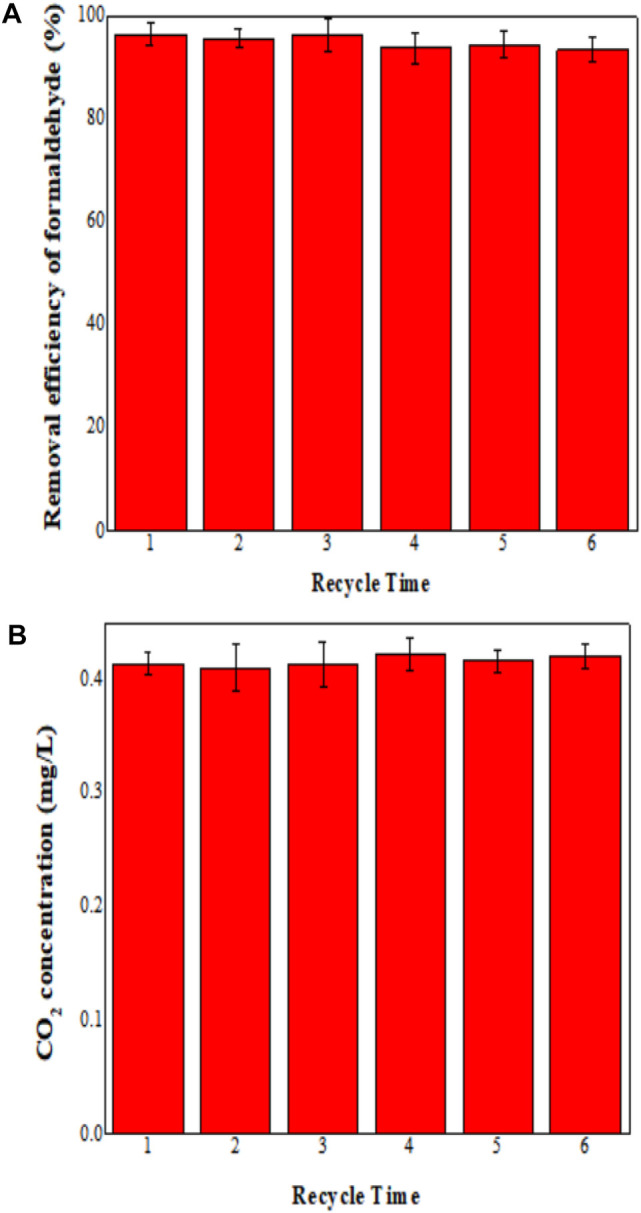
**(A)** Stability analysis of MoS_2_/OMC photocatalysts under repetitive experiments and **(B)** CO_2_ concentration under repetitive experiments.

The stability of the MoS_2_/OMC photocatalyst was an important index to evaluate its practical value, and the stability performance of MoS_2_/OMC materials is shown in Figure 7A. The degradation efficiency of formaldehyde still remained over 90%, and there was no obvious reduction after six cycles of testing, which proved that the MoS_2_/OMC photocatalyst could keep its high mechanical strength, good stability, and easy to recycle. This was mainly because the hollow spherical MoS_2_ was assembled into an orderly structure of OMC. This structure of MoS_2_/OMC materials not only maintained high activity and high specific surface area of the nanosheets but also greatly increased the number of edge active sites. At the same time, the structure was stable and easy to recycle. Therefore, it effectively overcame the defect of agglomeration of nanomaterials. The photocatalytic reactor containing MoS_2_/OMC photocatalyst was highly active under the optimum operating conditions (formaldehyde concentration for formaldehyde removal was 1.0 mg/m^3^, and photocatalysts amount was 0.03 g/m^3^). To evaluate the photocatalytic performance of MoS_2_/OMC, the photodegradation of HCHO into CO_2_ and H_2_O under visible light irradiation was employed. As shown in Figure 7B, the amount of the degraded HCHO was almost the same as generated CO_2_. The results showed that the MoS_2_/OMC photocatalyst had high activity and good stability.

## Conclusion

In this work, MoS_2_/OMC composite photocatalyst was successfully synthesized by using the hydrothermal method, and the structure, morphology, and electrochemical properties of the MoS_2_/OMC composite photocatalyst was characterized by SEM, XRD, and BET equation and BJH method. Besides, the experimental results showed that MoS_2_/OMC photocatalyst had the higher formaldehyde removal efficiency than the pure MoS_2_ and OMC. The higher photocurrent of the MoS_2_/OMC photocatalyst was ascribed to the longer electron–hole recombination rate. Moreover, the radical quenching experiments showed that formaldehyde removal was mainly attributed to the hydroxyl radicals and superoxide anion. The recycle testing showed that the MoS_2_/OMC photocatalyst had high activity and good stability.

## Data Availability

The original contributions presented in the study are included in the article/Supplementary Material; further inquiries can be directed to the corresponding author.
